# A low-dose, 6-week bovine colostrum supplementation maintains performance and attenuates inflammatory indices following a Loughborough Intermittent Shuttle Test in soccer players

**DOI:** 10.1007/s00394-017-1401-7

**Published:** 2017-03-11

**Authors:** Yiannis Kotsis, Anastasia Mikellidi, Cleopatra Aresti, Eleni Persia, Aristomenis Sotiropoulos, Demosthenes B. Panagiotakos, Smaragdi Antonopoulou, Tzortzis Nomikos

**Affiliations:** 1 Department of Nutrition and Dietetics, Faculty of Health Sciences and Education, Harokopio University, Eleftheriou Venizelou 70, 17671 Athens, Greece; 2 Faculty of Physical Education and Sport Sciences, National and Kapodistrian University of Athens, Athens, Greece

**Keywords:** Bovine colostrum, Exercise-induced muscle damage, Loughborough Intermittent Shuttle Test, Soccer, Inflammation, Squat jump

## Abstract

**Purpose:**

The aim of the study was to investigate the effect of a 6-week, low-dose bovine colostrum (BC) supplementation on exercise-induced muscle damage (EIMD) and performance decline in soccer players following the Loughborough Intermittent Shuttle Test (LIST) during a competitive season period.

**Methods:**

In a double-blind, randomized, placebo-controlled design, two groups of soccer players were allocated to a 3.2 g/day of whey protein (WP, *N* = 8) or BC (*N* = 10) and performed a pre- and a post-supplementation LIST. Maximum isometric voluntary contraction, squat jump (SQJ), countermovement jump, muscle soreness, blood cell counts, creatine kinase (CK), C-reactive protein (CRP) and interleukin-6 (IL-6) were monitored for 2, 24, 48, 72 h post-LIST.

**Results:**

LIST induced transient increases in leukocytes, granulocytes, CK, muscle soreness, CRP, IL-6 and declines in lymphocytes and performance indices. Supplementation resulted in a faster recovery of SQJ, CK and CRP compared to pre-supplementation kinetics (trial × time: *p* = 0.001, 0.056, 0.014, respectively) and lower incremental area under the curve (iAUC) for IL-6, only in the BC group [pre-: 31.1 (6.78–46.9), post-: 14.0 (−0.16 to 23.5) pg h/ml, *p* = 0.034]. Direct comparison of the two groups after supplementation demonstrated higher iAUC of SQJ [WP: −195.2 (−229.0 to (−52.5)), BC: −15.8 (−93.2 to 16.8) cm h, *p* = 0.034], a trend for lower iAUC of CK in the BC group [WP: 18,785 (4651–41,357), BC: 8842 (4807–14,802) U h/L, *p* = 0.081] and a significant intervention × time interaction for CRP (*p* = 0.038) in favor of BC.

**Conclusions:**

Post-exercise EIMD may be reduced and performance better maintained by a low dose of BC administration following LIST in soccer players.

**Electronic supplementary material:**

The online version of this article (doi:10.1007/s00394-017-1401-7) contains supplementary material, which is available to authorized users.

## Introduction

Elite-level soccer games are characterized by high-intensity intermittent physical activity. The repetitive and eccentric nature of soccer movements (accelerations, decelerations, kicking, jumping, and tackling) can cause muscle damage, accompanied by the leakage of muscle enzymes and other functional proteins in the circulation, oxidative stress, inflammation and decline in muscle performance [1, 2]. The clinical phenotype of post-match muscle damage can strongly impair the recovery process [3]. Taking into account the congested schedule of elite soccer players (2 or even 3 matches per week) the recovery period is not optimal for the restoration of pre-match exercise capacity and performance, while it may increase the risk for muscle injuries [4].

Since the extent of muscle damage can determine the duration of the recovery period, several strategies, aiming to attenuate muscle damage and accelerate the recovery period, are under research. However, the majority is adopting physiotherapeutic and training modalities like stretching, cryotherapy, active recovery, sleeping, massage, electrical stimulation and compression garments [5], while the nutritional or supplementation interventions are much less studied under real post-soccer match conditions. Interventions including the administration of creatine [6], antioxidant vitamins [7, 8], astaxanthin [9], beta-alanine [10], omega-3 fatty acids [11] are tested for their ability to reduce exercise-induced muscle damage (EIMD) or to increase exercise performance after a soccer match or a simulation of a soccer match. However, the results so far are inconclusive concerning the ability of supplements to improve the recovery process [5].

Bovine colostrum (BC) is the first milk produced by the mammary glands of the mother during the final days of pregnancy and the first days postpartum (1–4 days). It is as rich in carbohydrates, proteins, fats and micro-nutrients (minerals, trace elements) as the regular milk, but it additionally contains high amounts of oligosaccharides, growth factors, antimicrobial agents and immunoregulatory compounds [12]. It is therefore a natural multi-ingredient supplement with potential pleiotropic properties. Previous studies focused, mainly, on its effect on exercise performance, body composition [13], immune system and its ability to counteract upper respiratory tract infections [14]. However, the doses of BC in these studies were relatively high (20–60 g/day). So far, the ability of BC to enhance the recovery after a high-intensity soccer match is implied only indirectly through its anabolic properties [15], its buffering capacity [16] and its ability to attenuate post-exercise intestinal permeability [17] and oxidative stress [18]. In order to shed more light on this issue, aim of the present study was to investigate the effect of a low-dose BC supplementation on EIMD and performance in soccer players, during their competitive season, following the Loughborough Intermittent Shuttle Test (LIST) which simulates the activity pattern of a real soccer match [19].

## Materials and methods

### Study design

The study had a double-blind, randomized, placebo-controlled, parallel group, design. Participants initially completed a pre-supplementation LIST (LIST 1) and muscle damage indices were monitored for 72 h post-exercise. Then, they randomly allocated to the whey protein (WP) or BC group, received the respective supplements for 6 weeks and repeated the LIST protocol (LIST 2) by the end of the intervention period. The muscle damage and performance indices were again monitored for 72 h.

A week before LIST 1 subjects visit the laboratory for the preliminary screening, cardiopulmonary assessment and familiarization of all experimental procedures and LIST protocol. On the day of LIST 1, players arrived at the laboratory between 07:00 and 08:00 am after an overnight fast. A resting blood sample was taken and muscle strength, jumping performance, perceived muscle soreness and body composition were assessed. Afterwards, subjects consumed a standard meal consisting of two whole grain bread slices, a slice of smoked turkey fillet, 30 g of yellow Swiss cheese and a 240 ml of fresh orange juice (giving approximately 397 kcal; 59 g carbohydrates, 11 g fat, 15 g protein). EIMD biochemical markers, through venous blood samples, muscle performance and perceived muscle pain were evaluated 2, 24, 48 and 72 h post-LIST. Participants who successfully completed LIST 1 were randomly allocated to WP or BC group and received 3.2 g/day of WP or BC, respectively, for 6 weeks. By the end of the supplementation period all volunteers repeated the experimental protocol (LIST 2) and evaluations with the same order and time intervals.

**Fig. 1:**
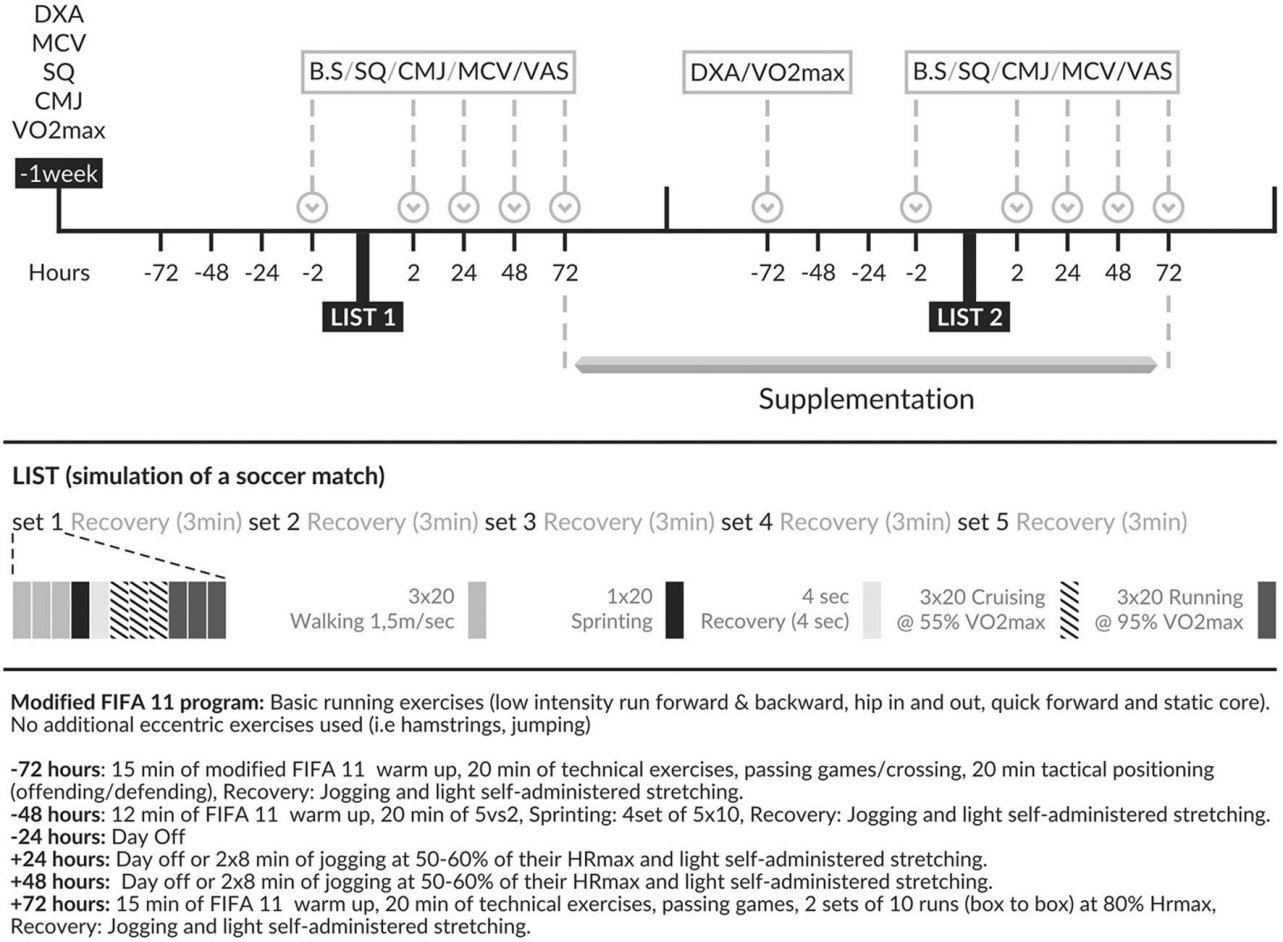
Schematic representation of the experimental protocol

The experimental protocol took place during the competitive period of the soccer season. During the whole protocol period, subjects were instructed to keep their usual eating habits and to restrain from any additional vitamin, antioxidant and performance-enhancing dietary supplementation or recovery treatment. Compliance to the protocol was checked with 24-h recalls. Subjects were also instructed to abstain from exhaustive exercise for 72-h pre- and post-LISTs and followed a standardized training regimen. During the whole supplementation period, with the exception of the 72-h pre- and post-LIST periods, the players were engaged in their habitual soccer training routine that included 4–5 training sessions and one official match per week. A detailed depiction of the experimental design is shown in Fig. 1.

### Ethics

The protocol was approved by the Ethical Committee of Harokopio University. All procedures performed in studies involving human participants were in accordance with the ethical standards of the institutional and/or national research committee and with the 1964 Helsinki declaration and its later amendments or comparable ethical standards. Informed consent was obtained from all individual participants included in the study.

### Subjects

**Fig. 2:**
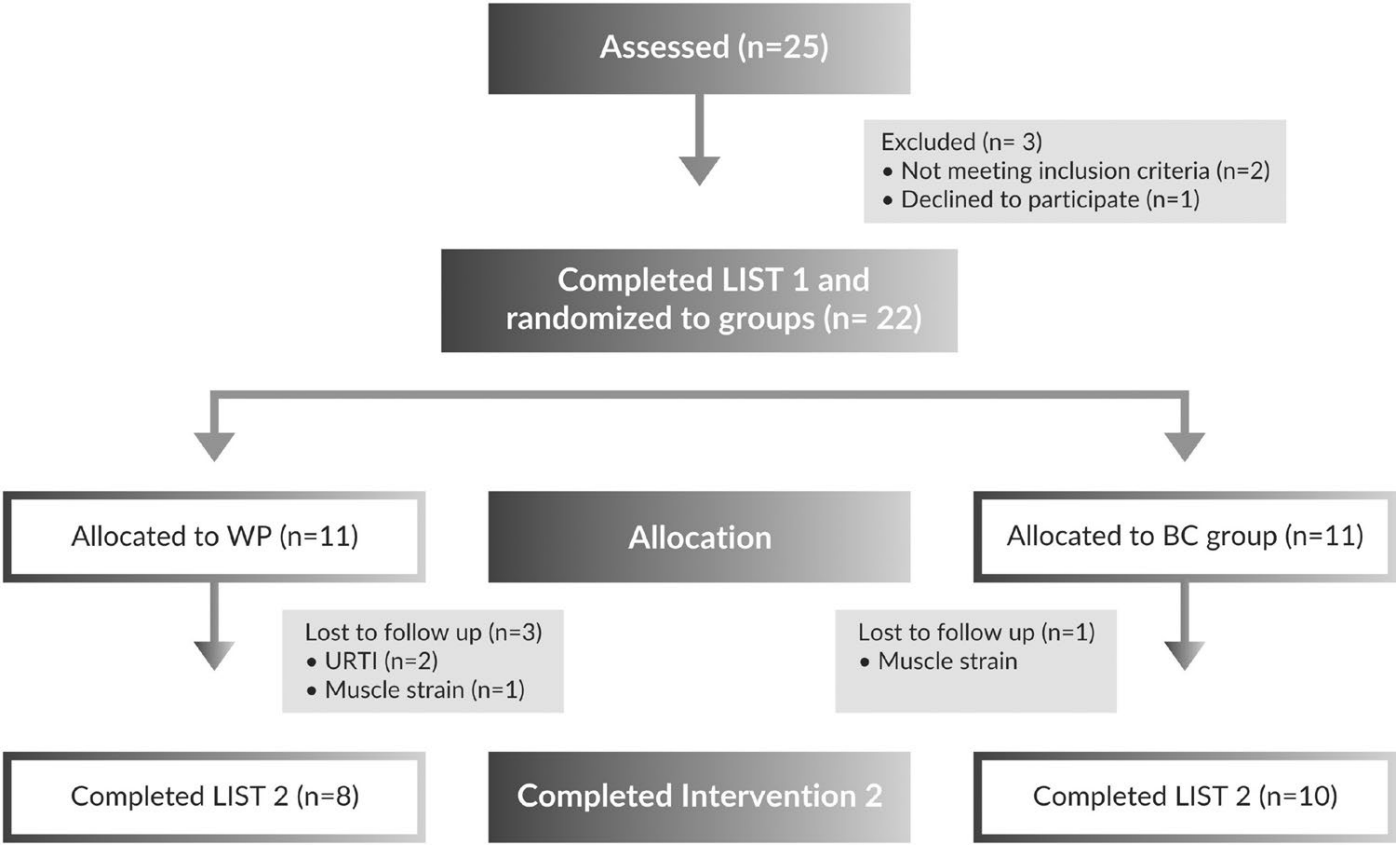
Flowchart of volunteers’ recruitment

For the purposes of the study 25 soccer players from the third and fourth division of the Greek National league were initially recruited (Fig. 2). Exclusion criteria were medical treatment, history of cardiovascular or any other inflammatory disease, cold or flu, acute respiratory infection, dental problems, renal/hepatic abnormalities, lactose intolerance and recent history of muscle injuries. Three of them were excluded from the study either because they did not meet the inclusion criteria or they declined participation. The rest of them (*N* = 22) completed the pre-supplementation LIST (LIST 1) and allocated to either the placebo group (WP, *N* = 11) or the bovine colostrum group (BC, *N* = 11). Three volunteers from the WP group and one volunteer from the BC group could not complete the intervention protocol due to upper respiratory tract infection and muscle strain. At the end, eight volunteers of the WP group and ten volunteers of the BC group completed the post-supplementation LIST (LIST 2) (Fig. 2). The WP group consisted of one defender, five midfielders, one full back/winger and one central attacker while BC group consisted of four defenders, four midfielders, one full back/winger and one central attacker.

### Sample size determination

To evaluate standardized differences in CK levels equal to 20%, a sample size of 10 participants per each group was considered adequate to achieve statistical power >0.90 at significance level of *p* < 0.05 of two-sided hypotheses. Despite the fact that we finished with less subjects in the WP group (*n* = 8), the working sample was also adequate to achieve statistical power equal to 90% having the aforementioned conditions.

### Randomization

A random sequence of numbers (i.e., 01001…) was created by an algorithm in MS Excel. Participants were allocated to BC (if 0) or WP (if 1).

### Supplementation

Participants of the BC group received 3.2 g/day (8 capsules × 400 mg of BC per capsule) of a commercially available bovine colostrum containing 378 kcal, 67 g protein, 17 g carbohydrates and 4.7 g fat per 100 g (Colostrum Compact, LR Health and Beauty Systems, Germany) while the WP group received 3.2 g/day of commercially available whey protein (8 capsules × 400 mg of WP per capsule) without flavoring and sweeteners (Ener Zona Proteine Whey 90%, Italy) containing 369 kcal, 90 g protein, 1 g carbohydrates and 0.5 g fat per 100 g. The whey protein was encapsulated in capsules, provided by LR, which were identical with the ones containing BC. The participants were advised to consume 4 capsules twice per day, half an hour before breakfast and dinner and were given 3 identical 56-capsule boxes containing either BC or WP, enough for a specific supplementation period of 3 weeks. By the end of the third week subjects were given the last four capsule boxes containing enough capsules for the remaining 3 weeks plus the 4 days of LIST 2. Then they were asked to record their daily supplementation consumption and bring back the boxes with the remaining capsules inside them. A 85 and 82% adherence was recorded for the WP and BC group, respectively.

### Loughborough Intermittent Shuttle Test

The 90-min shuttle run test was conducted according to Thompson et al. in an artificial surface soccer pitch [20]. Volunteers were required to run between two lines, 20 m apart, at various speeds dictated by an audio signal based on the velocities corresponding to their individual 55 and 95% of *V*O_2max_. The protocol consisted of 5 blocks of continuous running separated by 3 min of passive recovery for a total duration of 90 min. Heart rate was recorded via telemetry at 5-s intervals (Polar Team2 Pro, Polar, Finland) and averaged for each set (Fig. 1).

### Anthropometric and body composition measurements

Body weight and standing height were measured in light clothing, without shoes using a digital scale (Seca 861; Hamburg, Germany) with an accuracy of 0.1 kg and a stadiometer (Seca Leicester Height Measure; Seca, Vogel & Halke) to the nearest 0.1 cm. Body composition was assessed by DXA (Lunar, Corporation, Brussels, Belgium) set at medium speed and according to the manufacturer’s instructions [21].

### Nutritional assessment

Dietary intake before supplementation was assessed by three 24-h recalls which were collected the week before LIST 1 (two weekdays and one weekend). Similarly, dietary intake after supplementation was assessed by three more 24-h recalls, collected the week before LIST 2. The type of foods consumed (e.g., in terms of fat-content, brand name, constituents of mixed dishes, etc.), and the quantities or volumes were recorded in detail, using common household or other measures. Data from recalls were analyzed for their energy, macro- and micronutrient content by the Nutritionist Pro, version 2.2 software (Axxya Systems-Nutritionist Pro, Stafford, TX, USA), using a hand-coding procedure. The Nutritionist Pro food database was expanded by adding analyses of traditional Greek food and recipes.

### Cardiopulmonary assessment

The players performed an incremental (0.5 km/h increase each 1 min step) treadmill (Technogym Runrace, Gambettola, Italy) test until voluntary exhaustion to determine maximal oxygen uptake (*V*O_2max_), maximal heart rate (Polar Electro, Finland) and running speeds corresponding to 55 and 95% of *V*O_2max_. Expired respiratory gas fractions were measured using an open circuit breath-by-breath automated gas-analysis system (Vmax 229D, Sensormedics, Yorba Linda, CA).

### Maximum isometric voluntary contraction (MIVC)

During their first visit at the laboratory all subjects performed a specific submaximal protocol on the isokinetic dynamometer (BIODEX System 3 Pro, NY, USA) to familiarize with the isokinetic device and test procedure. Before muscle function measurements, subjects perform a standardized warm-up consisting of 5-min period on a cycle ergometer (Monark E-824) with a fixed load corresponding to 2% of body weight. MIVC of the knee extensors was measured at a knee joint angle of 90° (0° = full knee extension). The subjects were instructed to kick as hard and fast as they could for five maximal repetitions lasting 5 s separated by 60 s of rest. The three MIVC trials were averaged together to give the score for that particular measurement period.

### Jump performance

Squat jump (SQJ) and countermovemt jump (CMJ) tests were conducted using a photocell device (Optojump, Microgate, Italy) according to Bosco et al. [22]. Subjects warmed up for 5 min of on a stationary bicycle and performed 2 sets of 6 reps of submaximal SQJ and CMJ jumps by bending the knees to a preferred starting push-off position for CMJ and from a fixed knee bent at 90° for SQJ. For both jumping techniques hands were placed on hips. Then 3 maximal jumps were executed for each technique. A minimum of 2-min rest was allowed between jump trials. The highest vertical jump (cm) and the longest flying time (s) were registered and used for data analyses.

### Perceived muscle soreness (PMS)

Subjects self-reported muscle soreness by performing 3 full squat movements and passive stretching of specific muscles (quadriceps, hamstrings, abductors, adductors and gastrocnemius). We used a visual analog scale (VAS) from 0 (absence of soreness) to 10 (extremely sore) and a visual hologram of human muscles [20] where subjects pointed at the specific muscle area and rated the soreness.

### Blood sampling and biochemical assays

Venous blood samples were obtained from each subject. Complete blood count was determined in EDTA anticoagulated whole blood with a Mindray BC-3000 hematology analyzer (Mindray, Shenzhen, P.R. China). The activity of creatine kinase (CK) was determined spectrophotometrically by a commercially available kit (Biosis, Athens, Greece) modified for 96-well plates according to manufacturer’s instructions. High-sensitivity CRP and IL-6 were determined in serum by commercially available ELISA kits (Quantikine, R&D Systems, Abingdon, UK).

### Statistical analysis

Quantitative variables are reported as median with interquartile range (25th percentile–75th percentile) and qualitative, as frequencies. In order to estimate the response of each variable across the whole recovery period incremental, areas under the characteristic curve (iAUCs) were calculated for each variable by the trapezoid rule. Due to the small sample size and the skewed distribution we used the non-parametric Mann–Whitney test to compare: (a) the pre-LIST (baseline) values of WP and BC pre-supplementation and post-supplementation and (b) the iAUCs of WP and BC groups’ pre-supplementation and post-supplementation. A non-parametric analysis for repeated measures (Friedman test) was also applied to identify significant changes of VAS indices over time. Post hoc analysis with Wilcoxon signed-rank tests was conducted with a Bonferroni correction applied due to multiple comparisons, resulting in a significance level set at *p* < 0.01. Comparison of the response curves (WP LIST 1 vs WP LIST 2, BC LIST 1 vs BC LIST 2, WP LIST 1 vs BC LIST 1 and WP LIST 2 vs BC LIST 2) were performed using a two-way (trial × time) repeated-measures ANOVA, using log-transformed variables to reduce non-uniformity of error. ANOVA models incorporated a Greenhouse–Geisser correction for multisample asphericity. Any significant main effects identified in the ANOVA were further analyzed for post hoc paired t tests with Holm–Bonferroni correction. All statistical calculations were made with SPSS version 21.0 (Statistical Package for Social Sciences, SPSS, Chicago, Illinois, USA).

## Results

The WP and BC groups shared similar anthropometric, training, performance, hematological and biochemical pre-exercise characteristics that did not change after the 6-week supplementation period (Table 1). Dietary analysis did not reveal any significant differences in macronutrient intake between LIST 1 and LIST 2, neither for the WP group, nor for the BC group. In detail, energy intake for WP group was 2633 ± 472 vs 2711 ± 728 kcal (protein: 17.8 ± 3.7 vs 18.3 ± 3.6%, carbohydrates: 44 ± 8.4 vs 38.9 ± 7.8%, fat: 38.9 ± 8.9 vs 38.1 ± 5.6%) and for BC group 2676 ± 562 vs 2582 ± 372 kcal (protein: 19.3 ± 3.4 vs 18.2 ± 5.1%, carbohydrates: 43 ± 7.4 vs 43.9 ± 71.6%, fat: 36.4 ± 5.4 vs 38.6 ± 56.6%) for LIST 1 vs LIST 2, respectively. No differences between the dietary intake of WP and BC were also observed before LIST 1 or LIST 2.

**Table 1:** Baseline, pre-LIST, characteristics of volunteers in the WP and BC groups

	LIST 1		LIST 2	
	WP	BC	WP	BC
Age (years)	21.5 (19.5–22.2)	22.0 (19.5–23.2)	21.5 (19.5–22.2)	22.0 (19.5–23.2)
BMI (kg/m^2)^	22.9 (22.2–23.4)	23.6 (22.8–24.1)	23.0 (22.3–23.6)	23.3 (22.8–23.9)
Training (min/week)	505 (397–637)	570 (417–600)	520 (367–621)	552 (400–588)
Total fat (% of BW)	13.4 (10.8–15.2)	14.1 (10.5–14.4)	13.4 (11.0–14.7)	14.7 (11.4–15.9)
*V*O_2max_ (mL/kg/min)	51.4 (49.5–54.3)	53.7 (51.8–54.3)	51.0 (49.2–54.0)	52.3 (50.4–54.1)
MI VC (Nm)	321 (269–337)	349 (274–375)	315 (242–374)	299 (260–365)
CMJ (cm)	34.1 (31.3–37.8)	36.5 (32.3–38.6)	33.6 (32.8–38.0)	34.8 (32.7–37.5)
SQJ (cm)	30.8 (29.4–35.4)	34.3 (30.4–36.6)	32.0 (30.1–36.4)	33.1 (29.5–34.6)
White blood cells (10^3^/μL)	6.2 (5.0–6.9)	5.5 (4.9–5.9)	6.2 (5.7–6.8)	5.3 (4.8–6.3)
Lymphocytes (10^3^/μL)	2.2 (2.0–2.6)	2.2 (1.9–2.4)	2.5 (2.0–2.8)	2.4 (1.8–2.6)
Neutrophils (10^3^/μL)	3.5 (2.3–4.2)	2.6 (2.4–3.5)	3.6 (3.1–6.1)	2.9 (2.2–4.2)
Erythrocytes (10^6^/μL)	5.19 (4.92–5.44)	5.06 (4.65–5.77)	5.02 (4.87–5.39)	5.13 (4.71–5.47)
Hemoglobin (g/dL)	15.3 (14.4–16.6)	15.5 (14.3–15.9)	15 (14.1–15.6)	14.7 (13.9-15.05)
Platelets (10^3^/μL)	222 (199–249)	226 (192–238)	210 (166–286)	199 (180–249)
Creatine kinase (U/L)	168 (102–396)	152 (100–249)	143 (79–358)	174 (108–249)
C-reactive protein (mg/dL)	0.27 (0.18–0.41)	0.40 (0.21–0.57)	0.29 (0.18–0.90)	0.46 (0.33–0.65)
IL-6 (pg/mL)	0.61 (0.40–0.86)	0.62 (0.42–1.0)	0.65 (0.42–1.11)	0.78 (0.55–0.96)

Results are given as median (interquartile range). No significant differences were observed between the pre-LIST 1 values of WP and BC or between the pre-LIST 2 values of WP and BC (Mann–Whitney *U* test). No significant differences were observed between the pre-LIST 1 and pre-LIST 2 values of WP group and between the pre-LIST 1 and pre-LIST 2 values of BC group (Wilcoxon signed-rank test)

**Fig. 3:**
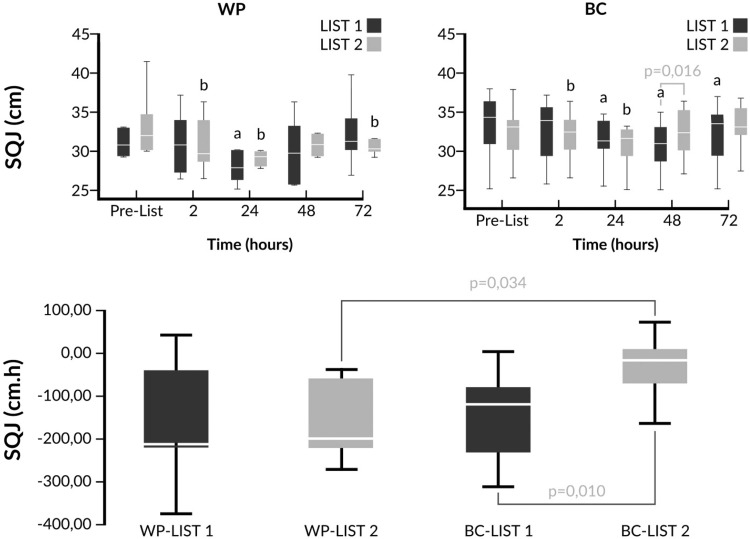
Changes of SQJ and comparison of iAUCs in response to LIST pre- and post-supplementation. ^a^ *p* < 0.05 vs pre-LIST 1 values, ^b^ *p* < 0.05 vs pre-LIST 2 values. SQJ values were log transformed prior to ANOVA analysis. WP LIST 1 vs WP LIST 2 (two-way ANOVA main effects): *p*(trial) = 0.751; *p*(time) ≤ 0.001; *p*(trial × time) = 0.190. BC LIST 1 vs BC LIST 2 (two-way ANOVA main effects): *p*(trial) = 0.893;* p*(time) <0.001; *p*(trial × time) = 0.001. WP LIST 1 vs BC LIST 1 (two-way ANOVA main effects): *p*(intervention) = 0.680; *p*(time) ≤ 0.001; *p*(intervention × time) = 0.207. WP LIST 2 vs BC LIST 2 (two-way ANOVA main effects): *p*(trial) = 0.819; p(time) ≤ 0.001;* p*(trial × time) = 0.065

**Fig. 4:**
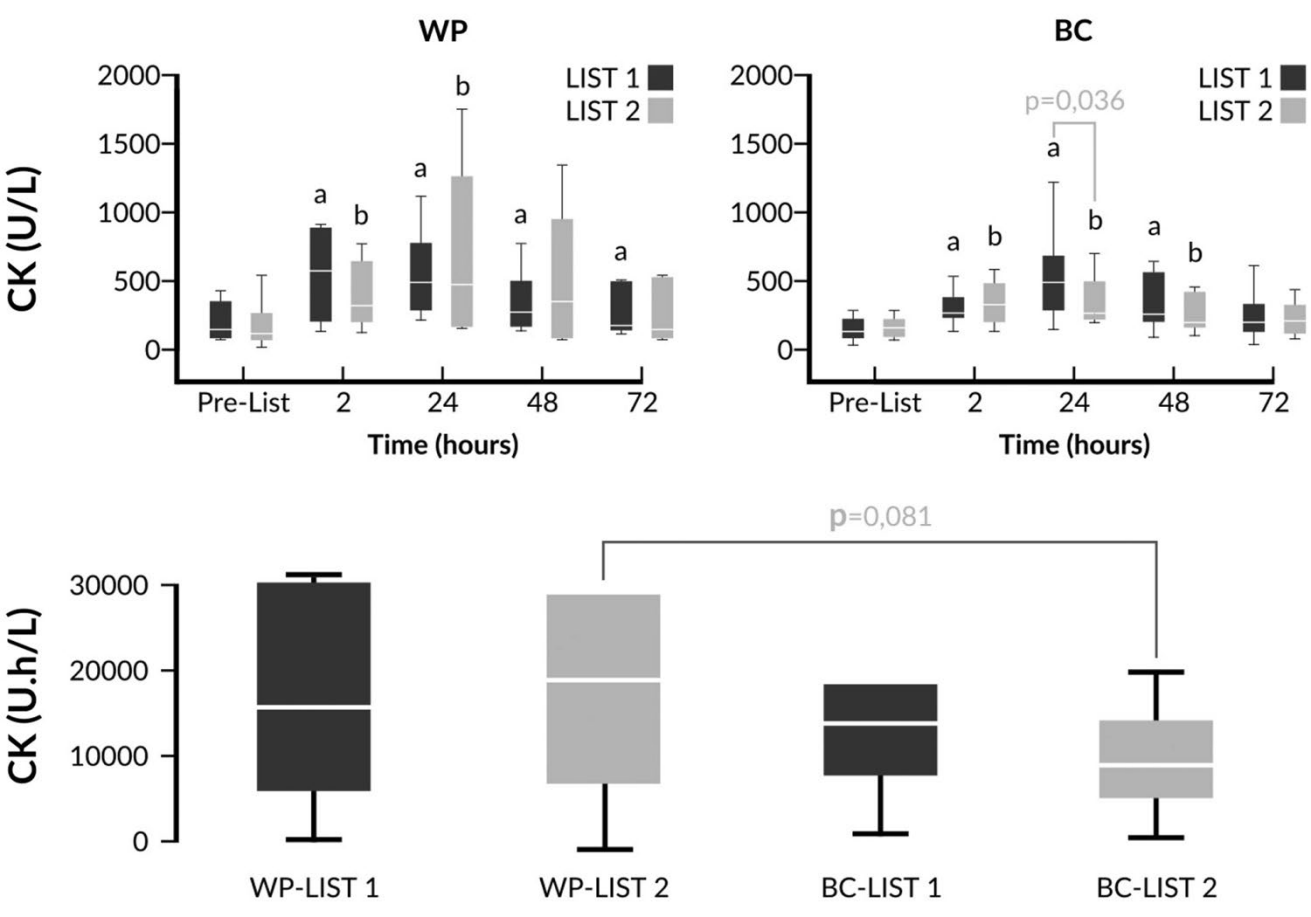
Changes of CK and comparison of iAUCs in response to LIST pre- and post-supplementation. ^a^ *p* < 0.05 vs pre-LIST 1 values, ^b^ *p* < 0.05 vs pre-LIST 2 values. CK and iAUC values were log transformed prior to ANOVA analysis. WP LIST 1 vs WP LIST 2 (two-way ANOVA main effects): *p*(trial) = 0.833; *p*(time)  ≤ 0.001; *p*(trial × time) = 0.743. BC LIST 1 vs BC LIST 2 (two-way ANOVA main effects): *p*(trial) = 0.799; p*(*time)  ≤ 0.001; *p*(trial × time) = 0.056. WP LIST 1 vs BC LIST 1 (two-way ANOVA main effects): *p*(intervention) = 0.492; *p*(time)  ≤ 0.001; p(intervention × time) = 0.527. WP LIST 2 vs BC LIST 2 (two-way ANOVA main effects): *p*(trial) = 0.671; *p*(time)  ≤ 0.001; *p*(trial × time) = 0.246

**Fig. 5:**
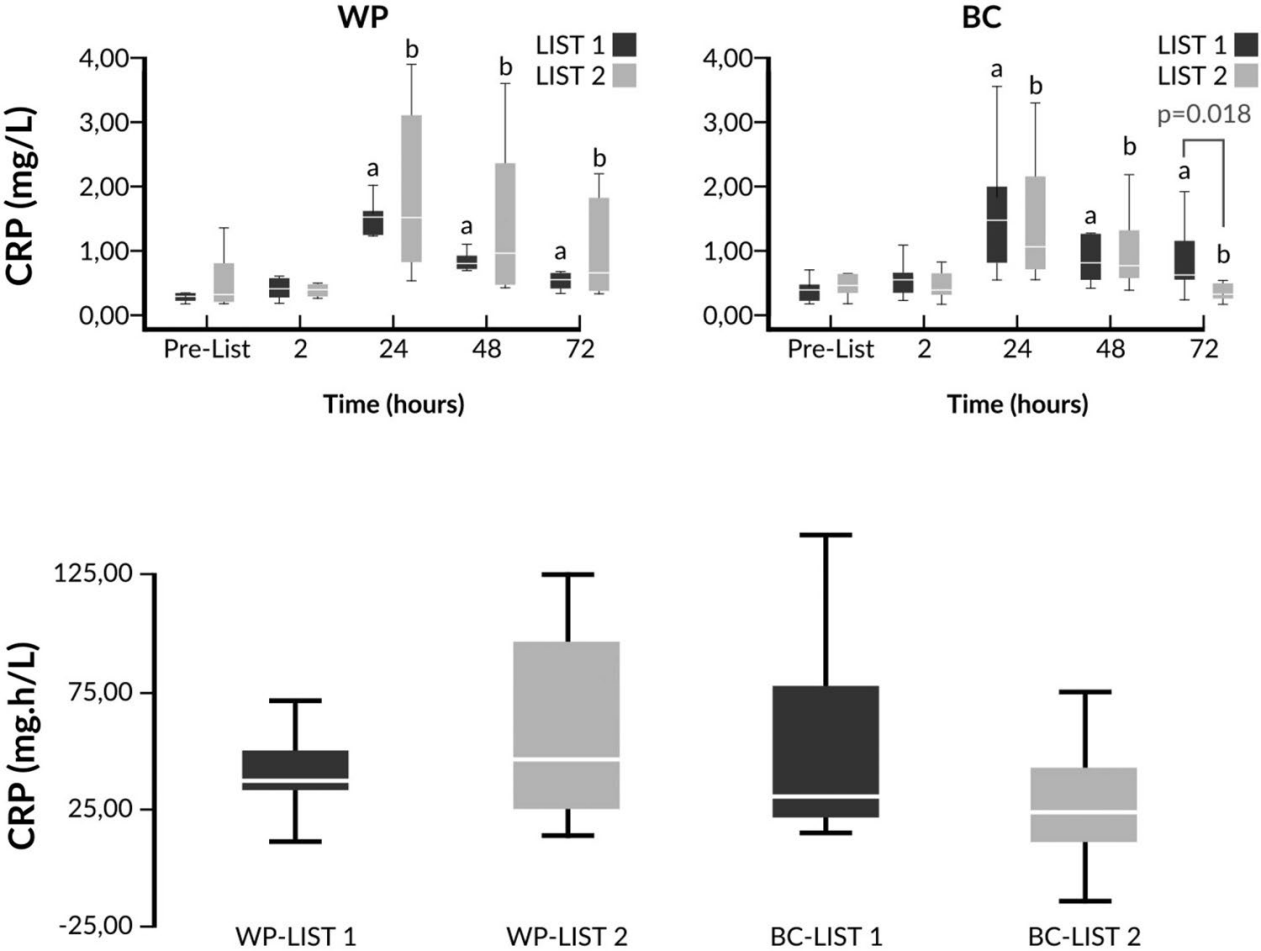
Changes of CRP and comparison of iAUCs in response to LIST pre- and post-supplementation. ^a^ *p* < 0.05 vs pre-LIST 1 values, ^b^ *p* < 0.05 vs pre-LIST 2 values. CRP and iAUC values were log transformed prior to ANOVA analysis. WP LIST 1 vs WP LIST 2 (two-way ANOVA main effects): *p*(trial) = 0.632; *p*(time)  ≤ 0.001; *p*(trial × time) = 0.663. BC LIST 1 vs BC LIST 2 (two-way ANOVA main effects): p(trial) = 0.544; *p*(time)  ≤ 0.001; *p*(trial × time) = 0.014. WP LIST 1 vs BC LIST 1 (two-way ANOVA main effects): *p*(intervention) = 0.417; *p*(time) ≤ 0.001; *p*(intervention × time) = 0.615. WP LIST 2 vs BC LIST 2 (two-way ANOVA main effects): *p*(trial) = 0.717; *p*(time) ≤ 0.001; p(trial × time) = 0.038

**Fig. 6:**
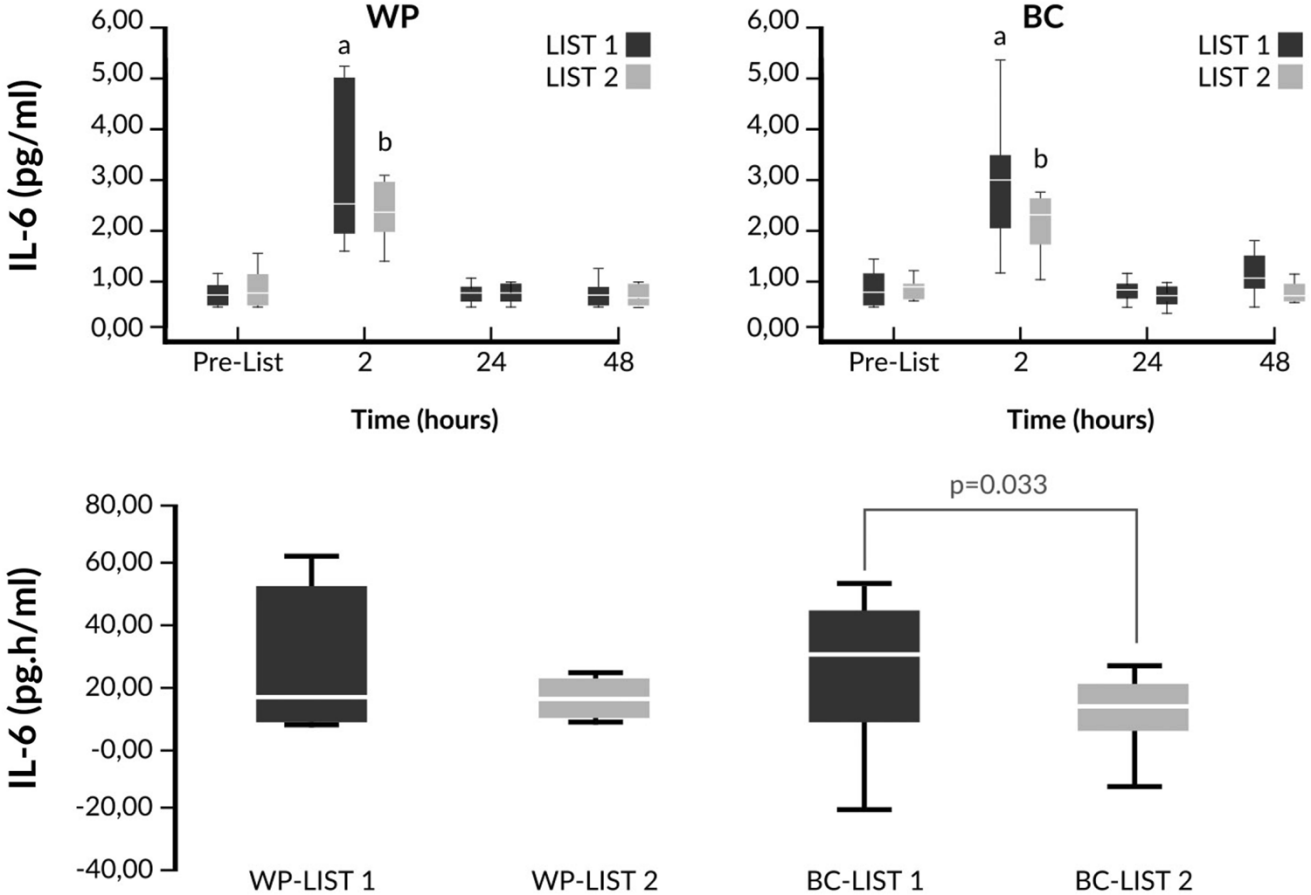
Changes of IL-6 and comparison of iAUCs in response to LIST pre- and post-supplementation. ^a^ *p* < 0.05 vs pre-LIST 1 values, ^b^ *p* < 0.05 vs pre-LIST 2 values. IL-6 and iAUC values were log transformed prior to ANOVA analysis. WP LIST 1 vs WP LIST 2 (two-way ANOVA main effects): *p*(trial) = 0.853; *p*(time)  ≤ 0.001; *p*(trial × time) = 0.529. BC LIST 1 vs BC LIST 2 (two-way ANOVA main effects): *p*(trial) = 0.445; *p*(time)  ≤ 0.001; p(trial × time) = 0.218. WP LIST 1 vs BC LIST 1 (two-way ANOVA main effects): *p*(intervention) = 0.302; *p*(time) ≤ 0.001; *p*(intervention × time) = 0.392. WP LIST 2 vs BC LIST 2 (two-way ANOVA main effects): *p*(trial) = 0.757; *p*(time)  ≤ 0.001; *p*(trial × time) = 0.447

The pre-supplementation kinetics of the measured variables (LIST 1) demonstrated either transient or sustained changes from baseline values in both groups indicating the ability of LIST to induce post-exercise hematological, biochemical and performance alterations (Tables 2, 3; Figs. 3, 4, 5, 6). Specifically, an acute but transient, twofold increase, of WBC is observed 2h post-LIST 1. The rapid elevation of circulating WBC is attributed to a ~2.5-fold increase of granulocytes although a significant 30% decline of lymphocytes was also observed at the same time point. The leukocyte levels returned to baseline values at 24 h and remained there up to 72 h post-LIST 1. Concerning the exercise performance indices, a sustained significant decline of all markers (MIVC, SQJ and CMJ) was observed from 2 to 48 h post-LIST 1. MIVC declined ~8 to 10% from 2 to 48 h, CMJ declined ~8 to 10% from 24 to 48 h while a 7–9% decline of SQJ was observed at 24–48 h. Moreover, a significant increase of perceived muscle pain in all muscle groups was evident, mainly at 24–48 h. The greater increase was found in knee extension and flexion muscles. Finally, CK values started to elevate 2-h post-LIST 1 but peaked at 24 or 48 h depending on the volunteer (3- to 4-fold increase). A delayed increase, at 24–48 h (fivefold), was demonstrated for CRP while the increase of IL-6 was acute showing a fourfold peak at 2h post-exercise and a return to basal levels at 24 h. The comparison of the iAUCs of all measured variables after LIST 1 demonstrated no significant differences between WP and BC groups indicating that the response to the LIST protocol, for all measured EIMD variables, was similar between the two groups (data not shown).

**Table 2:** Changes of hematological markers in response to pre-supplementation LIST 1 and post-supplementation LIST 2 in the WP (*N* = 8) and BC group (*N* = 10) group

	Intervention	Trial	Pre-LIST	2h	24h	48h	72h	iAUC	*p* values (trial; time; interaction) LIST 1 vs LIST 2	*p* values (intervention; time; interaction) WP LIST 2 vs BC LIST 2
WBC (10^9^/L)	WP	LIST 1	6.2 (5.0–6.9)	12.4^a^ (8.4–13.1)	6.0 (4.9–6.6)	5.8 (5.4–6.5)	5.8 (5.0–6.8)	53.2 (31.6–100.3)	0.992; <0.001; 0.304	0.606; <0.000; 0.072^2^
		LIST 2	6.2 (5.7–6.8)	12.0^a^ (9.6–13.4)	6.1 (5.0–8.3)	5.2 (4.8–6.4)	5.5 (4.8–6.9)	22.4 (−13.0 to 113.7)		
		*p* ^1^	NS	NS	NS	NS	NS	NS		
	BC	LIST 1	5.5 (4.9–6.0)	10.0^a^ (8.5–15.6)	5.9 (5.3–6.6)	5.7 (5.3–7.0)	5.7 (5.0–6.6)	94.8 (53.4–132.6)	0.817; <0.001; 0.617	
		LIST 2	5.3 (4.8–6.4)	10.5^a^ (7.3–11.4)	5.4 (4.8–6.6)	5.9 (5.2–7.2)	5.8 (4.8–7.4)	58.5 (13.4–84.2)		
		*p* ^1^	NS	NS	NS	NS	NS	NS		
Lym/cytes (10^9^/L)	WP	LIST 1	2.2 (2.0–2.6)	1.5^a^ (1.2–2.1)	2.3 (2.0–2.4)	2.1 (2.0–2.8)	2.2 (1.8–2.8)	−4.6 (−19.7 to 3.2)	0.358; <0.001; 0.271	0.521; <0.000; 0.056^2^
		LIST 2	2.2 (2.0–2.8)	1.5^a^ (1.2–1.9)	1.9 (1.7–2.6)	1.9 (1.6–2.4)	1.9 (1.6–2.2)	−36.7^b^ (−57.3 to (−17.3))		
		*p*	NS	NS	NS	NS	NS	NS		
	BC	LIST 1	2.2 (1.9–2.4)	1.7^a^ (1.5–1.8)	2.2 (1.9–2.40)	2.2 (1.9–2.4)	2.0 (1.6–2.4)	−6.6 (−22.6 to 8.3)	0.791; <0.001; 0.459	
		LIST 2	2.4 (1.8–2.6)	1.8 (1.6-2.0)	2.3 (1.6–2.4)	2.4 (1.8–2.6)	2.3 (1.5–2.5)	−11.2^b^ (−19.4 to 8.6)		
		*p*	NS	NS	NS	NS	NS	NS		
Gran/cytes (10^9^/L)	WP	LIST 1	3.5 (2.3–4.2)	10.7^a^ (5.7–1.8)	3.7 (2.7–3.9)	3.7 (2.8–3.8)	3.5 (2.6–3.7)	47.8 (39.6–88.9)	0.442; <0.001; 0.163	0.333; <0.001; 0.071^2^
		LIST 2	3.6 (3.1–6.1)	9.7^a^ (5.9–1.2)	3.9 (2.6-8.0)	2.8 (2.4–4.1)	3.4 (2.6–4.6)	28.2 (27.1–56.1)		
		*p*	NS	NS	NS	NS	NS	NS		
	BC	LIST 1	2.6 (2.4–3.5)	7.6^a^ (6.6–11.2)	3.4 (2.8–4.2)	3.2 (2.3–4.4)	3.2 (2.4–4.2)	61.2 (39.7–131.9)	0.736; <0.001; 0.467	
		LIST 2	2.9 (2.2–4.2)	8.1^a^ (4.9–8.8)	2.7 (2.2–4.4)	2.7 (2.6–3.9)	3.0 (2.0–4.0)	72.9 (13.2–98.2)		
		*p*	NS	NS	NS	NS	NS	NS		

Results are shown as median with interquartile range (25th percentile–75th percentile)

All variables (except for iAUCs) were log transformed prior to ANOVA analysis

WBC: WP LIST 1 vs BC LIST 1 (two-way ANOVA main effects): *p*(intervention) = 0.634; *p*(time) < 0.001; *p*(intervention × time) = 0.773

Lym/cytes: WP LIST 1 vs BC LIST 1 (two-way ANOVA main effects): *p*(intervention) = 0.566; *p*(time) < 0.001; *p*(intervention × time) = 0.773

Gran/cytes: WP LIST 1 vs BC LIST 1 (two-way ANOVA main effects): *p*(intervention) = 0.897; *p*(time) < 0.001; *p*(intervention × time) = 0.716

^1^ *p* values of paired *t* test analysis for the comparison of WP LIST 1 vs WP LIST 2 and BC LIST 1 vs BC LIST 2 at specific time points

^2^Two-way ANOVA main effects for WP LIST 2 vs BC LIST 2

^a^Significantly different from pre-LIST values (*p* ≤ 0.05) based on post hoc analysis

^b^Significant differences between WP LIST 2 vs BC LIST 2 based on Mann–Whitney test

**Table 3:** Changes of exercise performance indices in response to pre-supplementation LIST 1 and post-supplementation LIST 2 in the WP (*N* = 8) and BC intervention group (*N* = 10)

	Intervention	Trial	Pre-LIST	2h	24h	48h	72h	iAUC	*p* values (trial; time; interaction) LIST 1 vs LIST 2	*p* values (intervention; time; interaction) WP LIST 2 vs BC LIST 2
MΙVC (N m)	WP	LIST 1	321 (264–337)	294^a^ (275–305)	291^a^ (255–296)	290^a^ (254–312)	304^a^ (254–320)	−1990 (−2400 to (−534))	0.272; 0.001; 0.865	0.521; 0.135; 0.445^2^
		LIST 2	349 (269–375)	314^a^ (260–349)	327^a^ (273–344)	339 (273–347)	338 (278–343)	−1341 (−2334 to (−145))		
		*p* ^1^	NS	NS	NS	NS	NS	NS		
	BC	LIST 1	330 (246–384)	293 (221–363)	276^a^ (217–335)	279^a^ (216–348)	303 (219–363)	−1340 (−3572 to (−559))	0.617; 0.212; 0.055	
		LIST 2	299 (260–366)	288^a^ (247–344)	306 (292–332)	326 (256–360)	291 (265–356)	−630 (−1278 to 167)		
		*p* ^1^	NS	NS	NS	NS	NS	NS		
CMJ (cm)	WP	LIST 1	34.1 (31.3–37.8)	32.8 (30.7–36.8)	32.4 (29.4–36.5)	32.1 (29.9–36.2)	34.9 (30.4–37.2)	−52 (−301 to 2.9)	0.990; 0.004; 0.468	0.670; <0.001; 0.481^2^
		LIST 2	33.6 (32.8–38.0)	32.3^a^ (31.3–37.0)	30.8^a^ (29.9–33.2)	32.9^a^ (30.9–36.2)	32.9^a^ (31.4–37.7)	−143 (−154 to (−121))		
		*p*	NS	NS	NS	NS	NS	NS		
	BC	LIST 1	36.5 (32.3–38.6)	36.8 (31.2–38.0)	34.0^a^ (30.4–35.7)	34.0^a^ (30.2–36.4)	35.0 (30.0–37.4)	−140 (−167 to (−63))	0.798; <0.001; 0.136	
		LIST 2	34.9 (32.7–37.6)	34.4 (31.6–37.5)	34.8^a^ (30.4–35.3)	35.9 (32.9–36.9)	35.0 (31.8–36.9)	−43 (−145 to 20)		
		*p*	NS	NS	NS	NS	NS	NS		

Results are shown as median with interquartile range (25th percentile–75th percentile). All variables (except for iAUCs) were log transformed prior to ANOVA analysis

MIVC: WP LIST 1 vs BC LIST 1 (two-way ANOVA main effects): *p*(intervention) = 0.866; *p*(time) = 0.012; *p*(intervention × time) = 0.894

CMJ: WP LIST 1 vs BC LIST 1 (two-way ANOVA main effects): *p*(intervention) = 0.869; *p*(time) = 0.001; *p*(intervention × time) = 0.713

^1^ *p* values of paired *t* test analysis for the comparison of WP LIST 1 vs WP LIST 2 and BC LIST 1 vs BC LIST 2 at specific time points

^2^ Two-way ANOVA main effects for WP LIST 2 vs BC LIST 2

^a^Significantly different from pre-LIST values (*p* ≤ 0.05) based on post hoc analysis

In order to assess whether the 6-week supplementation with either WP or BC was able to change the volunteers’ response to the LIST protocol, we compared the kinetics of the measured variables pre- and post-supplementation (WP LIST 1 vs WP LIST 2 and BC LIST 1 vs BC LIST 2 and) for each group, separately. Specifically, we compared the absolute values and percent changes at each time point along with the iAUC of LIST 1 and LIST 2 for every volunteer in a pair-wise fashion. In addition, a direct comparison between groups was made before (WP LIST 1 vs BC LIST 1) and after supplementation (WP LIST 2 vs BC LIST 2).

Concerning the leukocyte counts no significant differences of WBC, granulocytes and lymphocyte kinetics were observed after LIST 2 compared to LIST 1, either in the WP or the BC group, indicating that neither WP nor BC supplementation were able to alter the leukocytes response to LIST (Table 2). Direct comparisons of WP vs BC before and after supplementation also showed no differences between groups in the exercise-induced changes of the hematological markers although a higher iAUC for lymphocytes (*p* = 0.017) was observed in the BC group compared to WP group after LIST 2 (Table 2).

Comparison of the VAS recordings at individual time points or as iAUC demonstrated no significant differences of delayed-onset muscle soreness in none of the five muscle groups between LIST 1 and LIST 2 in both groups with the exception of lower VAS recordings of the knee flexors after post-supplementation LIST 2 in the WP group (Table 4, Supplemental material).

When the performance responses were compared, similar declines for MIVC and CMJ were observed for LIST 1 and LIST 2 in both groups (Table 3). However, a faster recovery of the SQJ performance was observed only in the BC group which was reflected in a significant trial × time interaction (*p* = 0.001) between BC LIST 1 and BC LIST 2, a significant intervention × time interaction between WP LIST 2 and BC LIST 2 and a better SQJ performance 48h post-LIST 2 compared to post-LIST 1 in the BC group. Although absolute values could not show a significant trial effect and intervention effect, when ANOVA analysis was conducted with the % changes of SQJ values (data not shown) significant differences were observed between BC LIST 1 and BC LIST 2 (*p*trial = 0.048) and between WP LIST 2 and BC LIST 2 (*p*intervention = 0.010). In addition, less negative iAUCs were recorded for the nine out of ten volunteers in the BC group. This trend was not observed for the WP group (Fig. 3).

When the responses of the biochemical markers were compared between pre- vs post-supplementation LISTs, we observed a non-significant trend for lower elevations of CK activity post-LIST 2 compared to post-LIST 1 only in the BC group. This trend reached statistical significance 24 h after LIST (Fig. 4). A direct comparison between WP and BC after the supplementation demonstrated a trend for a lower iAUCs in the BC group compared to the WP group after LIST 2 (*p* = 0.081) (Fig. 4).

A significant time × trial interaction for CRP kinetics was observed only in the BC group without any significant trial effect (Fig. 5). Lower CRP values were observed at 72h after LIST 2 compared to LIST 1 only in the BC group. No significant difference between the iAUCs of the trials was found (Fig. 5). When ANOVA analysis was conducted with the % changes of CRP values (data not shown) significant differences were observed between BC LIST 1 and BC LIST 2 (*p* for trial = 0.038) and between WP LIST 2 and BC LIST 2 (*p* for intervention = 0.031).

Finally, significantly lower increments of IL-6 were observed in LIST 2 compared to LIST 1 only in the BC group when we compared the iAUCs (Fig. 6) and the percent changes from baseline 2h after LIST [LIST 1: 351% (243–601%); LIST 2: 254% (178–339%), *p* = 0.028].

## Discussion

The present study aimed to investigate the ability of a long-term, low-dose BC supplementation to enhance the recovery process of soccer players after a simulated soccer match (LIST) by alleviating EIMD symptoms. The outcomes of the study demonstrate a trend for BC supplementation to maintain performance (SQJ decline) and attenuate post-exercise increments of CRP, CK and IL-6 in contrast to WP supplementation.

LIST protocol is a well-established, soccer simulation protocol able to induce similar responses to a typical soccer match but in a more controlled fashion [19, 20]. For this reason, LIST has been widely used in supplementation studies which were mainly focused on the potential ergogenic effect in exercise performance [10, 23] fuel replenishment [24] and fluid balance [25]. On the other hand, only few supplementation studies have implemented LIST or other simulated soccer pattern activities [6, 26] and real soccer matches [8] to examine the alleviation of EIMD on clinical and biochemical features. Compared to other studies we adopted a relatively long-term (>4 weeks) and low-dose (3.2 g/day vs 20–60g/day in other studies) supplementation protocol where the efficacy of the supplementation was assessed in a repeated measures design. For the dosage quantity we decided that it should be close enough to the recommendations of the company (800 mg/day). We speculate that the high bioavailability of BC in combination with the high quality would be enough to modulate post-exercise kinetics. Despite WADA’s statement that “Colostrum is not prohibited per se, however it contains certain quantities of IGF-1 and other growth factors which are prohibited and can influence the outcome of anti-doping tests” (https://www.wada-ama.org/en/questions-answers/prohibited-list#item-388) it is unlikely that our dose scheme could elicit a positive doping test result. The study of Kuipers et al. clearly showed that a 20-fold higher supplementation dose than ours (60 g/day of BC powder), could not change blood IGF-I or IGF binding protein-3 levels and does not elicit positive results on drug tests [27].

In order to attenuate the high inter-individual variability of such protocols, we compare the response of the same volunteer to LIST pre- and post-supplementation. Moreover, for the simulation of real field settings we incorporated the experimental period during the in-season soccer period. Subjects were committed to a typical soccer training micro-cycle where a team trains on average >6 times and plays one official game per week. During the period of 3 days preceding and 4 days following the LIST, the training load was fixed and controlled by specific training instructions made by our team (Fig. 2). Considering the training background and the engagement in daily training and weekly soccer team routine, we assumed that the repeated bout effect would not be an issue in our experimental conditions.

Our LIST protocol induced similar changes to performance, biochemical and hematological markers of EIMD compared to other studies [1, 28, 29]. In contrast to other similar studies we estimated PMS separately in five leg muscle groups. Due to the eccentric nature of the LIST protocol, a significant increase of perceived muscle pain was observed in all muscle groups but was more pronounced in knee extensors and flexors since they receive the majority of the work load during the acceleration and decelerations phase. Especially the knee flexors contract eccentrically during soccer actions such as kicking, sprinting or changing of directions in order to decelerate thigh and leg movements prior to foot landing [30].

In agreement to our study, the presence of leukocytosis, immediately post-exercise, has also been observed after both LIST and soccer matches although our study showed in a more emphatic way that this leukocytosis is attributed to a dramatic increase of granulocytes, despite a concomitant decrease of lymphocytes 2h post-LIST. Similar acute neutrophilia and lymphopenia was observed after a soccer match in both male [31] and female players [32] while other studies were unable to observe this pattern [26, 33]. The main mechanism underlying the exercise-induced lymphopenia is the egress of certain lymphocyte subtypes (NK cells, γδ Tcells, CD8^+^ T cells) from the blood to peripheral tissues under the influence of glucocorticoids while lymphocyte apoptosis seems to be of minor importance for the observed lymphopenia [34].

Finally, post-exercise elevations of EIMD biochemical marker were in accordance with similar studies after both LIST [7, 28] and soccer protocols [1, 35, 36]. However, the magnitude of the CK increase was lower in our study compared to others [7, 28] probably because our volunteers were soccer players, therefore accustomed to the eccentric loads of LIST, especially since the experimental phase took place during the competitive period of the year. Similar CK increments were observed in the work of [3] where volunteers were also competitive soccer players in full training and match schedule.

Both LIST and real soccer matches have been previously used by research to assess the potential beneficial effect of dietary supplements on exercise performance of soccer players. However, studies investigating the ability of supplements to alleviate symptoms of EIMD are scarce and give contradictory results due to different methodological procedures, dose schemes and biochemical measurements. Supplementation with vitamin C [37], a mixture of antioxidant vitamins [7], beta-alanine [10] and green tea polyphenols [38] could not attenuate clinical and biochemical symptoms of EIMD after LIST or similar intermittent tests. On the other hand, DHA supplementation showed a beneficial effect on oxidative stress and inflammatory response after an acute exercise session in soccer players [11]. It is therefore obvious that dietary supplementation in soccer players has not been studied thoroughly and the supplements used until now are incapable of exerting protection against EIMD. This is probably because most of them contain one or few active ingredients with a specific action (e.g., radical scavenging activity), while EIMD is a multifactorial process.

On the other hand, BC is the only naturally produced multi-ingredient supplement. It contains a milieu of bioactive nutrients and microconstituents with antimicrobial and immunomodulatory properties mainly in the alimentary tract, such as lactoferrin, lactoperoxidases, lysozyme, IGF-1/2, peptides and oligosaccharides [14, 39]. The effect of BC supplementation on several aspects of exercise performance and body composition has been extensively studied before; however, the results are contradictory [13]. This can be mainly due to differences in the experimental design, source and composition of BC, dosage schemes and subject’s fitness level and training background. However, promising results were obtained when BC was studied for its ability to attenuate post-exercise immune suppression which is achieved through several mechanisms that include restriction of post-exercise decrements of IgG2 and salivary IgA and lysozyme [40, 41], neutrophils degranulation [40] and protection against exercise-induced gut hyper-permeability [17]. However, the efficiency of BC to protect against EIMD symptoms in soccer players has not been studied before although indirect evidences imply that BC may act as a recovery enhancer. Brinkworth et al. have shown that BC supplementation to active males (60 g/day, 8 weeks) is able to improve performance in an exercise bout 20 min after an initial bout of incremental running exercise to exhaustion [16]. A lower dose of BC (10 g/day, 5 weeks) was also able to reduce fatigue after a 5 day high intensity training period in highly trained cyclists [42]. The mechanisms underlying the ability of BC to affect the recovery process after one bout of exercise are still speculative. BC supplementation can increase resting levels of essential and branched-chain amino acids [43] which promote skeletal muscle synthesis and attenuate muscle damage [44]. Moreover, an animal model has shown that BC in the absence of exercise is able to lower lipid hydroperoxides, increase the specific activity of superoxide dismutase and increase total antioxidant capacity in muscle tissues of rats. When supplemented along with exercise it was able to attenuate exercise-induced increases of lipid hydroperoxides and xanthine oxidase and exercise-induced decreases of SOD and TAC [18]. Finally, BC is able to protect epithelial integrity and attenuate gut permeability partly by reducing temperature-induced apoptosis of epithelial cells and induction of the cytoprotective heat-shock proteins [17]. If similar mechanism were able to function in muscle tissues then they could favorably modulate EIMD. In contrast to other studies, we decided to proceed with a low-dose and relatively long-term dosage scheme which can be applied in real field settings, i.e., during the in-season soccer period.

The results of this study demonstrated the ability of BC supplementation, in contrast to WP supplementation, to attenuate post-LIST increments of CK, CRP and IL-6 accompanied by an improvement of SQJ decrements. BC could not significantly affect PMS, leukocyte counts, MICV, and CMJ kinetics, but instead it had a significant beneficial effect in exercise performance taking into account the improvement in SQJ. It should be mentioned that post-supplementation pre-LIST indices were not affected by BC or WP indicating that BC effects could only be attributed to changes in the post-exercise plasticity of the subject’s muscle and inflammatory responses. The greater susceptibility of SQJ to BC supplementation is in accordance with the results of Byrne et al. who found that SQJ performance was affected to a greater extent after an eccentric protocol than the CMJ [45]. These results suggest that the stretch–shortening cycle used in the CMJ possibly masks the negative performance effects associated with the exercise-induced muscle damage. Perhaps the SQJ is more susceptible to the inflammatory response accompanying EIMD than CMJ, which is under the influence of a more complex control and governed by advanced neuromuscular and proprioceptive biofeedback mechanisms. Under this perspective, the mild anti-inflammatory properties of BC may more strongly affect SQJ than CMJ.

It is tempting to speculate that BC, by attenuating muscle damage (CK and IL-6) can lead to a lower systemic inflammatory response. Whether this attenuation originates from a possible stabilization of muscle cells, which can induce a lower inflammatory response or the opposite, is not known. However, the anti-inflammatory properties of BC have been demonstrated both in cell and chronic inflammation models [14, 39], but these models do not necessarily share similar pathophysiological features with the exercise-induced inflammation. Recently, Appukutty and co-workers [18] demonstrated the ability of BC supplementation to protect against exercise-induced oxidative stress in rat muscles in a dose (per kg of body weight) similar to ours. In addition, recent studies in athletes have shown the ability of BC to attenuate gut permeability after heavy exercise in athletes [17]. It is therefore obvious that due to its multicomponent composition BC seems to exert pleiotropic beneficial action on post-exercise recovery. Whether these actions can be translated to long-term beneficial actions on recovery after intermittent exercise requires further mechanistic and supplementation studies.

Our study is the first soccer-specific research that tracks EIMD kinetics and incorporates professional and semi-professional soccer players accustomed to regular soccer eccentric training instead of amateurs and college students unaccustomed to eccentric exercise mode. In addition there was a high homogeneity between our subjects on their basic anthropometric, biochemical and exercise performance features (i.e., VO_2max_, BMI, training background). Another advantage of the experimental design was the implementation of the supplementation phase during the in season period of the professional league. The soccer training micro-cycle per se can produce an additional load on musculoskeletal system of the athlete and an added difficulty for the BC supplementation to overcome. We tried to simulate/mimic the real soccer field settings even though we knew that regular high-intensity soccer practice, with the cyclic pattern of concentric and eccentric extension/flexion of the knee per se, might alter the athlete performance decline. To overcome the latter, we decide to incorporate a specific training program 3 days pre- and 4 days post-LIST without any additional eccentric load. In addition, we choose functional testing like MICV, SQJ and CMJ that have minimum eccentric load and would not exaggerate or interfere with the EIMD symptoms caused by the LIST per se.

On the other hand, the low number of participants, the nature of the protocol which lacks soccer-specific movements like tackling or shooting, the limited range of functional tests, the use of maximal isometric strength of the knee extensors instead of knee flexors which are more susceptible to soccer eccentric movement pattern and the use of just one dose of BC supplementation are among the limitations of the study.

In conclusion, the LIST protocol can induce EIMD and performance decrements in soccer players. EIMD and performance following LIST intervention may positively be affected by a low-dose administration of BC in soccer players. Larger studies are needed to confirm the ability of bovine colostrum to improve recovery after a soccer game.

## Electronic supplementary material

Below is the link to the electronic supplementary material.

Supplementary material 1 (DOCX 27 KB)

## Abbreviations

iAUCs: Incremental area under the curve
BC: Bovine colostrum
CK: Creatine kinase
CMJ: Counter movement jump
CRP: C-reactive protein
EIMD: Exercise-induced muscle damage
IL-6: Interleukin-6
LIST: Loughborough Intermittent Shuttle Test
MIVC: Maximum isometric voluntary contraction
SQJ: Squat jump
VAS: Visual analog scale
WP: Whey protein
