# Supplementary material for: A low-dose, 6-week bovine colostrum supplementation maintains performance and attenuates inflammatory indices following a Loughborough Intermittent Shuttle Test in soccer players
Source: Eur J Nutr. 2017 Mar 11;57(3):1181–95. doi: 10.1007/s00394-017-1401-7 (PMC5861165; doi:10.1007/s00394-017-1401-7)
Supplement: Supplementary file 1 — Supplementary material 1 (DOCX 27 KB) [file 394_2017_1401_MOESM1_ESM.docx]

|  | **Intervention** | **Trial** | **Pre** | **2** | **24** | **48** | **72** | **iAUC** | **P^2^** |
| --- | --- | --- | --- | --- | --- | --- | --- | --- | --- |
| **VAS Knee Extensors**  **(cm)** | WP | LIST 1 | 0.0  (0.0-0.0) | **3.2^a^**  (1.7-4.7) | **5.5^a^**  (3.3-7.2) | **4.8^a^**  (3.0-8.0) | **2.5^a^**  (1.5-3.2) | 300  (227-438) | **0.001** |
|  |  | LIST 2 | 0.0  (0.0-0.0) | **3.0^a^**  (1.8-5.0) | **4.5^a^**  (2.8-7.0) | **6.0^a^**  (4.0-7.2) | **3.0^a^**  (1.5-4.0) | 337.0  (207-401) | **0.001** |
|  |  | p | NS | NS | NS | NS | NS | NS |  |
|  | BC | LIST 1 | 0.0  (0.0-0.0) | **3.5^b^**  (2.0-5.0) | **5.5^b^**  (4.7-7.8) | **6.2^b^**  (4.0-7.0) | **2.7^b^**  (2.0-5.0) | 366  (269-424) | **0.000** |
|  |  | LIST 2 | 0.0  (0.0-0.0) | **3.0^b^**  (2.5-4.2) | **6.0^b^**  (4.8-7.6) | **5.0^b^**  (3.5-7.0) | **2.0^b^**  (0.8-4.2) | 331  (231-421) | **0.000** |
|  |  | p | NS | NS | NS | NS | NS | NS |  |
| **VAS Flexors**  **(cm)** | WP | LIST 1 | 0.0  (0.0-0.0) | **5.0^a^**  (1.8-5.5) | **6.0^a^**  (2.8-7.2) | **7.0^a^**  (6.0-8.0) | **3.5^a^**  (1.5-5.5) | 383  (282-452) | **0.001** |
|  |  | LIST 2 | 0.0  (0.0-0.0) | **3.0^a^**  (1.0-4.5) | **4.0^a^**  (1.8-7.0) | **3.0^a^**  (1.8-5.2) | **2.0^a^**  (0.8-3.2) | **212^a^**  (114-362) | **0.002** |
|  |  | p | NS | NS | NS | **0.026** | NS | **0.002** |  |
|  | BC | LIST 1 | 0.0  (0.0-0.0) | **5.5^b^**  (2.5-4.2) | **7.0^b^**  (4.8-7.6) | **7.2^b^**  (3.5-7.0) | **4.5^b^**  (0.8-4.2) | 461  (374-522) | **0.000** |
|  |  | LIST 2 | 0.0  (0.0-0.0) | **5.0^b^**  (4.0-6.2) | **7.5^b^**  (6.0-8.2) | **6.2^b^**  (4.0-7.0) | **3.0^b^**  (1.8-4.2) | 377  (303-465) | **0.000** |
|  |  | p | NS | NS | NS | NS | **0.016** | NS |  |
| **VAS Adductors**  **(cm)** | WP | LIST 1 | 0.0  (0.0-0.0) | **2.0^a^**  (0.7-3.2) | **3.5^a^**  (1.0-5.5) | **3.5^a^**  (0.7-6.2) | 0.5  (0.0-2.2) | 177  (64-342) | **0.003** |
|  |  | LIST 2 | 0.0  (0.0-0.0) | **2.0^a^**  (1.0-2.5) | **2.5^a^**  (2.0-5.2) | **1.5^a^**  (1.0-5.2) | 0.5  (0.0-3.0) | 123  (91-315) | **0.001** |
|  |  | p | NS | NS | NS | NS | NS | NS |  |
|  | BC | LIST 1 | 0.0  (0.0-0.0) | **4.5^b^**  (2.0-7.0) | **5.0^b^**  (2.5-7.2) | **3.5^b^**  (1.8-4.2) | **0.5^a^**  (0.0-3.5) | 264  (153-364) | **0.000** |
|  |  | LIST 2 | 0.0  (0.0-0.0) | **4.2^b^**  (1.0-6.2) | **5.5^b^**  (2.0-8.0) | **4.0^b^**  (1.8-7.0) | **2.0^b^**  (0.8-4.2) | 296  (106-421) | **0.000** |
|  |  | p | NS | NS | NS | NS | NS | NS |  |
| **VAS Abductors**  **(cm)** | WP | LIST 1 | 0.0  (0.0-0.0) | **1.5^a^**  (0.8-2.2) | **1.5^a^**  (0.8-6.2) | 0.5  (0.0-4.7) | 0.0  (0.0-1.5) | 64  (20-320) | **0.014** |
|  |  | LIST 2 | 0.0  (0.0-0.0) | **1.0^a^**  (1.0-3.2) | **2.0^a^**  (1.8-3.0) | **1.5^a^**  (1.0-3.2) | 0.5  (0.0-1.2) | 100  (91-150) | **0.002** |
|  |  | P | NS | NS | NS | NS | NS | NS |  |
|  | BC | LIST 1 | 0.0  (0.0-0.0) | **3.0^b^**  (1.5-3.2) | **3.5^b^**  (1.8-5.0) | **2.0^b^**  (0.8-3.5) | 0  (0.0-1.6) | 177  (103-254) | **0.000** |
|  |  | LIST 2 | 0.0  (0.0-0.0) | **2.0^b^**  (1.0-4.2) | **3.5^b^**  (1.8-5.2) | **2.5^b^**  (1.0-3.8) | **1.0^a^**  (0.0-2.2) | 183  (103-244) | **0.000** |
|  |  | p | NS | NS | NS | NS | NS | NS |  |
| **VAS Gas/nius**  **(cm)** | WP | LIST 1 | 0.0  (0.0-0.0) | **2.0^a^**  (0.8-3.0) | **2.5^a^**  (1.7-4.2) | 1.5  (0.0-3.8) | 0  (0.0-2.5) | 117  (43-253) | **0.002** |
|  |  | LIST 2 | 0.0  (0.0-0.0) | **2.5^a^**  (0.8-6.2) | 1.5  (0.0-6.8) | 1.5  (0.0-6.3) | 1.0  (0.0-4.5) | 106  (18-434) | **0.009** |
|  |  | p | NS | NS | NS | NS | NS | NS |  |
|  | BC | LIST 1 | 0.0  (0.0-0.0) | **2.5^b^**  (1.8-4.6) | **3.5^b^**  (1.8-7.2) | **3.0^b^**  (2.0-3.8) | **1.5^a^**  (0.0-3.1) | 200  (142-301) | **0.000** |
|  |  | LIST 2 | 0.0  (0.0-0.0) | **2.5^b^**  (1.5-6.0) | **3.5^b^**  (2.8-5.6) | **4.0^b^**  (2.0-5.0) | **2.0^b^**  (0.8-3.2) | 230  (159-351) | **0.000** |
|  |  | p | NS | NS | NS | NS | NS | NS |  |

**Table 4.** Changes of VAS in response to pre-supplementation LIST 1 and post-supplementation LIST 2 in the WP (N=8) and BC group (N=10) group.

Results are shown as median with interquartile range (25th percentile-75th percentile)

^1^ p values for paired t-test (Wilcoxon signed-rank test) between LIST 1 and LIST 2

^2^ p values for Friedman test analyses within treatment groups

^a^ Significantly different from pre-LIST values (0.01<p<0.05) based on post-hoc Wilcoxon signed-rank tests

^b^ Significantly different from pre-LIST values (p<0.01) based on post-hoc Wilcoxon signed-rank tests
